# Clinical outcomes after hip arthroscopy in acetabular dysplastic patients, previously treated with periacetabular osteotomy: a minimum of two-year follow-up data from the Danish Hip Arthroscopy Registry

**DOI:** 10.1093/jhps/hnae015

**Published:** 2024-04-08

**Authors:** Bjarne Mygind-Klavsen, Bent Lund, Torsten Grønbech Nielsen, Martin Lind

**Affiliations:** Department of Orthopedics, Aarhus University Hospital, Palle Juul-Jensens Boulevard 99, Aarhus N 8200, Denmark; Department of Orthopedics, Horsens Regional Hospital, Sundvej 25, Horsens 8700, Denmark; Department of Orthopedics, Aarhus University Hospital, Palle Juul-Jensens Boulevard 99, Aarhus N 8200, Denmark; Department of Orthopedics, Aarhus University Hospital, Palle Juul-Jensens Boulevard 99, Aarhus N 8200, Denmark

## Abstract

Periacetabular osteotomy (PAO) is the treatment of choice in dysplastic acetabulum. Due to continued symptoms, 2–11% of these patients require an additional hip arthroscopy. The purpose of this study was to report clinical outcome after a minimum of 2-year follow-up of additional hip arthroscopy after PAO with data from Danish Hip Arthroscopy Registry. Inclusion criteria in the study cohort were PAO surgery resulting in an additional hip arthroscopy procedure. The cohort was evaluated according to the surgical findings and patient-related outcome measures (PROMs) pre-operatively and at 2-year follow-up. A total of 287 patients were included in the study cohort. PROMs improved significantly in all subscales from pre-operatively to 2-year follow-up in the study cohort. According to PROM subscales, 47.8–57.6% and 25.2–38.2% achieved Minimal Clinical Important Difference and Patient Acceptable Symptom State, respectively. This study demonstrates, in PAO-treated patients, significant PROM improvements after additional hip arthroscopy. Unfortunately, only ∼50% and 30% achieved Minimal Clinical Important Difference and Patient Acceptable Symptom State, respectively.

## INTRODUCTION

Standard treatment for symptomatic hip dysplasia has for several years been the periacetabular osteotomy (PAO), as described by Ganz *et al*. [1]. The purpose of the osteotomy is to reorientate the acetabulum and thereby unload the labrum on the acetabular rim and resolve symptoms from concomitant intra-articular (IA) damage. This procedure has demonstrated good long-term hip survivorship in a Danish hip dysplasia cohort [2]. In that study, which included more than 1100 patients, PAO preserved 4 of 5 hip joints after 14 years, and furthermore, proved that PAO was a safe procedure with low major complication rates (1.1% required surgical intervention) and good subjective outcomes. In that cohort, an additional hip arthroscopy was performed in 11.1% (*n* = 154) of the cases. The indications for additional hip arthroscopy in that population were femoral osteochondroplasty, synovitis, cartilage injury and labral repair or resection. The risk of additional hip arthroscopy after the PAO procedure has been reported between 2% and 11% [3–5]. Hartig-Andreasen *et al*. [6] prospectively investigated and compared two patient groups, isolated PAO versus PAO and additional arthroscopy, regarding patient-reported outcome. The patients treated with additional arthroscopy demonstrated inferior outcomes for the combined procedure, and therefore additional arthroscopy in this patient cohort was not recommended in Denmark at that time.

The purpose of this study was to report clinical outcomes after hip arthroscopy in a dysplastic population, previously treated with PAO and a minimum of 2-year follow-up with data collected from the Danish Hip Arthroscopy Register (DHAR).

## MATERIALS AND METHODS

The DHAR is a web-based registry approved by the Danish Health Authorities, J.nr. 2012–58-0006 in 2012. The structure of this prospective hip arthroscopy database has been published [7]. All hospitals and private orthopedic surgeons, who perform hip arthroscopy, are encouraged to report surgical data which includes perioperative radiological data, intraoperative surgical techniques and IA pathologies. Since 2012, DHAR has registered more than 7700 hip arthroscopy procedures as published in the 2021 annual report [8].

### Patients

DHAR was assessed for eligible patients treated with PAO prior to hip arthroscopy.

Inclusion criteria, defining the study cohort, were patients treated with PAO surgery and afterwards, due to IA symptoms, needing an additional hip arthroscopy procedure. This procedure was not planned when patients were referred to PAO surgery and was performed at any time point after the PAO procedure. Exclusion criteria were other non-PAO patients treated with hip arthroscopy or other hip-related surgery, previous hip conditions such as Legg–Calvé–Perthes disease and avascular necrosis.

**Fig. 1.**
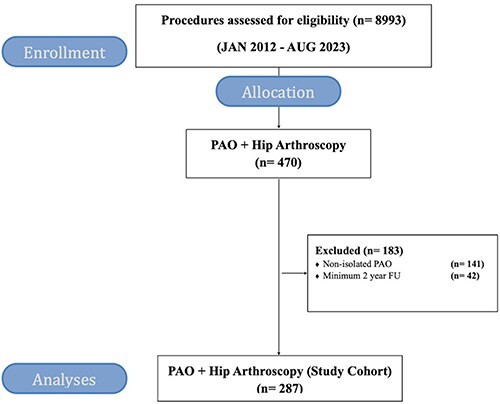
Consort flowchart of the eligible patients and how the study cohort was generated.

Patient selection is illustrated in Fig. 1. The male:female ratios were 10:90 in the study cohort. The average age was 29 years, with a range between 15 years and 62 years. Among the included patients in the study group, 35 patients received an additional hip-related surgical procedure within the 2-year observation period. These additional surgeries consisted of 10 revision hip arthroscopies with labrum repair procedures, 11 patients received a total hip replacement and 14 patients received other small hip surgeries. None of the PAO patients received the hip arthroscopy in combination with the PAO procedure.

### Outcome evaluation

DHAR provides information regarding patient demographics such as age and gender. Several PROMs, consisting of European Quality of Life–5 dimensions (EQ-5D) [9], Hip Sports Activity Scale (HSAS) validated and recommended for use in femoroacetabular impingement syndrpme (FAIS) populations [10], Pain Numeric Rating Scale (NRS rated pain during rest and after 15 min of walking) and the Copenhagen Hip and Groin Outcome Score (HAGOS) [11, 12] were submitted online to DHAR by included patients. HAGOS consists of six subscales, in total 37 questions assessing symptoms, pain, function in daily living, function in sport and recreation, participation in physical activities and hip and/or groin-related quality of life, are aimed for young to middle-aged adults undergoing non-surgical treatment or hip arthroscopy [12].

Radiological and surgical data and measurements are reported into the registry by the surgeon. Radiological data consist of: Wibergs lateral centre-edge (CE) angle, Tönnis acetabular index (AI) angle, lateral joint space width (JSW) at the lateral sourcil, alpha angle and the presence of posterior wall sign, crossover sign and prominent ischial spine sign. The surgical technique data include description of procedures by anatomical location: labral procedures (resection, repair or reconstruction), cartilage procedures (debridement, reinsertion or microfracture), bony procedures (acetabuloplasty and femoroplasty) and extraarticular procedures (capsular closure and tenotomy).

In DHAR, cartilage injuries are classified according to International Cartilage Repair Society (ICRS) [13] on the femoral head and modified Beck’s cartilage classifications [14–16] on the acetabular side.

MCID was determined by using the 0.5 SD of the baseline values for the HAGOS subscales as used by Thorborg *et al*. [17].

Patient Acceptable Symptom State (PASS) was determined according to cut-off values, as the data have been published recently based on a Danish FAIS cohort study. These PASS cut-off values have been estimated using HAGOS. The cut-off values in the six HAGOS subscales were: pain (68.75), symptoms (62.50), activity of daily living (ADL) (82.50), sport/rec (60.94), physical activity (PA) (43.75) and quality of life (QoL) (42.50), respectively [18].

### Statistical analysis

Dichotome and categorical data were presented as numbers (*n*) with percent (%). Continuous data were presented as mean with 95% confidence intervals (95% CIs) if data were normally distributed or mean with SD.

Pearson’s chi-squared and Fischer’s exact tests were used to compare dichotome and categorical data, depending on the numbers of data. Student’s *t*-test was used to compare continuous data. *P* values <0.05 were considered to be statistically significant. Statistical analysis was performed in Stata17 (StataCorp LP, TX, USA).

## RESULTS

### Patient selection and demographics

Figure 1 illustrates the patient selection process. A total of 8993 procedures were retrospectively identified through DHAR and of these, 470 procedures were performed in patients with previously PAO and therefore eligible for inclusion in the study group. Due to inclusion and exclusion criteria as mentioned above, 287 patients were included in the study group and 183 were excluded. Demographics and radiological measurements are illustrated in Table I.

**Table I.** Preoperative demographics and radiological data. Age is presented as median value with range, radiological measurements as mean (95% CI), numbers and (percentages)

	*Study cohort (PAO)*
*N*	287
Age [median (range)]	29 (15–62)
Gender (male:female)	10:90
Radiological measurements [mean (95% CI)]	
CE angle	32.2 (31.6–32.8)
Alpha angle	66.0 (64.4–67.9)
Tönnis AI angle	3.7 (3.2–4.3)
JSW (*n* (%)]	
JSW <3.0 mm	6 (2%)
JSW 3.1–4.0 mm	79 (28%)
JSW > 4.0 mm	202 (70%)
Ischial spine sign [*n* (%)]	38 (13%)
Crossover sign [*n* (%)]	70 (37%)
Posterior wall sign [*n* (%)]	33 (13%)

### Surgical hip arthroscopic procedures

The major surgical procedures are illustrated in Table II. A high proportion of the patients (76.2%) received a combined correction of both cam and pincer morphologies. Regarding labral procedures, repair was performed in almost all cases (92.8%). The treatment of cartilage injuries consisted mainly of cartilage debridement. Of the extra-articular procedures, psoas tenotomy was performed in 11.4% of the patients and capsular closure was performed in 45.7% of the patients. Fifty-seven patients received additional PAO screw removal in combination with their hip arthroscopy procedure.

**Table II.** Major IA and extraarticular surgical procedures performed

	*Study cohort (PAO)*
Mean operation time (min)	67 ± 30
Mean traction time (min)	40 ± 20
Surgical procedures—IA	
Bony procedures	*n = 269*
Femoral head–neck osteochondroplasty	229 (85.1%)
Acetabuloplasty/acetabular rim-trimming	245 (91.1%)
Isolated pincer resection	40 (14.9%)
Isolated cam resection	24 (8.9%)
Combined cam and pincer resection	205 (76.2%)
Labrum procedures	*n = 264*
Labrum resection	19 (7.2%)
Labrum repair	245 (92.8%)
Labrum reconstruction	0 (0%)
Cartilage procedures	*n *= 202
Cartilage debridement	123 (60.9%)
Cartilage resection femoral head	6 (3.0%)
Cartilage resection acetabulum	68 (33.6%)
Microfracture femoral head	1 (0.5%)
Microfracture acetabulum	4 (2.0%)
Surgical procedures—Extraarticular	*n = 140*
Psoas tenotomy	16 (11.4%)
Capsular closure	64 (45.7%)
Fjernelse af AO-skruer	57 (40.7%)
Fjernelse af forkalkning	2 (1.4%)
Partiel AIIS Resection	1 (0.7%)

### Cartilage injury

Table III illustrates the distribution of surgeon reported cartilage injuries in the study cohort. The proportion of high-grade femoral head cartilage injury, ICRS 2–4, was found in 26.7%, whereas 80.4% of the patients demonstrated high-grade acetabular cartilage injury, modified Becks 2–4.

**Table III.** Distribution of reported cartilage injury grade (ICRS and modified Becks) and estimated area (cm^2^) of injury in numbers and (percentages)

	*Study cohort (n = 255)*
Femoral head	
ICRS 0–1	187 (73.3%)
ICRS 2–4	68 (26.7%)
Area = 0 cm^2^	164 (64.3%)
Area < 1 cm^2^	31 (12.2%)
Area 1–2 cm^2^	33 (12.9%)
Area > 2 cm^2^	27 (10.6%)
Acetabulum	
Modified Becks 0–1	50 (19.6%)
Modified Becks 2–4	205 (80.4%)
Area = 0 cm^2^	6 (2.3%)
Area < 1 cm^2^	115 (45.1%)
Area 1–2 cm^2^	107 (42.0%)
Area > 2 cm^2^	27 (10.6%)

### Subjective outcomes

Table IV summarizes the subjective outcome scores and mean improvements. At the 2-year follow-up all subjective outcomes demonstrated significantly higher scores with score improvements of 12–16 points. The range in the HAGOS subscales varied between 15.9 and 49.0 pre-operatively versus 30.6–63.6 post-operatively.

**Table IV.** Subjective preoperative outcomes in the cohort and improvements 2 years after surgery. All values are listed as mean ± SD. A P-value of <0.05 is considered a statistically significant difference

	*Preoperative*	*2 year follow-up*	
*N*	*(n = 195)*	*(n = 131)*	*Sign. diff*.
HAGOS			
Pain	43.6 ± 16.4	59.6 ± 22.3	<0.001
Symptoms	41.9 ± 14.3	53.4 ± 21.0	<0.001
ADL	49.0 ± 20.8	63.6 ± 24.5	<0.001
Sport&rec	28.2 ± 18.4	43.1 ± 27.5	<0.001
PA	15.9 ± 19.0	30.6 ± 30.2	<0.001
QoL	24.4 ± 14.3	39.1 ± 23.6	<0.001
HSAS	2.1 ± 1.3	2.8 ± 1.8	<0.001
NRS pain rest	46.5 ± 22.8	31.2 ± 25.5	<0.001
NRS pain activity	63.1 ± 21.7	40.5 ± 29.8	<0.001
EQ-5D	0.58 ± 0.20	0.68 ± 0.24	<0.001

The percentages of patients achieving MCID and PASS according to HAGOS subscales are summarized in Table V. In the study cohort, 47.8–57.6% of the patients achieved MCID in HAGOS subscales. Regarding HSAS and EQ-5D 39.6% and 38.0% of the patients achieved MCID respectively.

**Table V.** The percentages of patients achieving MCID and PASS in HAGOS sub-scales. A P-value of <0.05 is considered as a statistically significant difference

	*MCID*	*PASS*
	*Study cohort (n = 92)*	*Study cohort (n = 131)*
HAGOS subscale		
Pain	53 (57.6)	47 (35.9)
Symptoms	44 (47.8)	45 (34.3)
ADL	44 (47.8)	35 (26.7)
Sport	47 (51.1)	33 (25.2)
PA	44 (47.8)	43 (32.8)
QoL	49 (53.3)	50 (38.2)

When measuring PASS according to cut-off values, the percentages of patients achieving PASS in the study cohort ranged from 25.2% to 38.2%. The lowest percentages were seen in HAGOS_ADL_ and HAGOS_Sport_, the highest were seen in HAGOS_Pain_ and HAGOS_QoL_.

## DISCUSSION

The primary purpose of this study was to report patient-reported outcomes with a minimum of 2-year follow-up of additional hip arthroscopy after PAO. This study demonstrates on average a significant improvement after a hip arthroscopy in all subjective outcomes measured, but only half had measureable symptom improvement and only 25-40% reached an acceptable symptom state.

The results of the current study demonstrated improved outcomes in this subpopulation undergoing hip arthroscopy after a PAO procedure and are in opposition to earlier Danish studies in this population. Hartig-Andreasen [19] demonstrated limited clinical benefit with no reduction in pain level and lack of improvements in subjective patient-reported outcomes. However, there are some differences between their study and the current study. At that time, Hartig-Andreasen used modified Harris Hip Score and Hip Outcome Score, since HAGOS had not yet been developed. HAGOS might be more suitable in this patient population [20, 21]. The previous study, also presented a low number of patients in combination with low data completeness, potentially making the current study more valid. Furthermore, it is possible that the study by Hartig-Andreasen might have resulted in surgeon awareness and changed the indications and contraindications before offering these patients hip arthroscopy after PAO surgery, especially regarding data on JSW (<3 mm) and Tönnis Grade (>2).

In terms of clinically relevant improvement in subjective outcome, the current study demonstrates that approximately half of the patients with continued symptoms after PAO achieved a clinically relevant improvement after a hip arthroscopic procedure at 2-year follow-up. When interpreting MCID, health care practitioners should be aware of the fact, that MCID gives a general information on the effect of the treatment and in patients terms, the percentage of patients that are ‘getting better’ after the treatment, and not necessarily the percentage of patients that are ‘feeling good’ [17]. For this purpose, the PASS has been recommended. When measuring PASS according to these cut-off values, as performed in the current study, the percentage of patients in the study cohort achieving PASS ranged from 25.2% to 38.2%. The lowest percentages were seen in HAGOS_ADL_ and HAGOS_Sport_, the highest were seen in HAGOS_Pain_ and HAGOS_QoL_.

Currently, it is evident that PAO surgery in an important number of cases results in a positive impact on life quality and may prevent or delay the need of total hip replacement in the hip dysplastic patient. Recently, a Danish Registry study demonstrated a 14-year survival rate of 80%. That study also reported a need for additional hip arthroscopy in 11% of the cases due to IA symptoms [2]. The arthroscopic procedures performed in this group were not reported and likewise, predictors of/or risk of additional procedures, were not described. Beaulé *et al*. [22] reported a higher alpha angle to be associated with lower outcomes, but identified no other demographic or radiographic variables that were associated with lower patient-reported outcomes or re-operations. Hartig-Andreasen *et al*. [6] prospectively investigated risk factors, in dysplastic patients undergoing PAO-surgery (*n* = 90), and the need for additional hip arthroscopy. A total of 26 patients underwent additional arthroscopic treatment and they found that mild dysplasia (defined as CE angle from 20 to <25°), preoperative crossover sign (X-ray) and detached labrum (MR-Arthrogram) were risk factors for additional arthroscopic treatment in their cohort. Therefore, whether to perform PAO correction concomitant with hip arthroscopy is not fully investigated and further studies are needed. Perhaps, restriction in hip motion after PAO correction or radiological signs, such as CE angle, alpha angle or crossover signs might indicate the need for additional hip arthroscopy.

To clarify on this topic, whether to perform concomitant hip arthroscopy and PAO or isolated PAO, a randomized trial is currently ongoing to provide more information regarding best treatment strategy in this patient cohort [23]. When comparing the results from the current study with studies where PAO was performed in combination with hip arthroscopy, Maldonado *et al*. [24] (16 patients) demonstrated significant mid-term (5 year) outcome improvements in PROMs and VAS pain. Furthermore, 50–75% of patients reached PASS and 78.6–81.3% reached the MCID in the two measured PROMs, respectively. A recent study by Edelstein *et al*. [25] consisting of 70 procedures, where hip arthroscopy was performed in combination with PAO demonstrated similar results in terms of improvements in PROMs and reaching the MCID after a mean of 6.5 years follow-up. Seventy-six percent of the hips were preserved (were not converted to total hip arthroplasty (THA) and did not have persistent symptoms) but 21% of the patients experienced persistent symptoms and 3% were converted to THA during the follow-up period. They found that progression to THA or persistent symptomatic hip was associated with lower baseline PROM score. Their study cohort was a selected cohort of dysplastic patients consisting of 22% of their total PAO cases. The patients were included if the clinical history and examination findings were consistent with IA symptoms (specifically, symptoms of intermittent sharp groin pain in combination with a positive anterior impingement test) and visualization of labral detachment from the acetabular rim or a focal cartilage defect on MRI. Adhering to the selection process, hip arthroscopy was subsequently recommended before PAO during the same operative setting. A recently published systematic review by Lee *et al*. [26] supports the treatment of dysplastic patients with concomitant hip arthroscopy and PAO. They documented favorable outcomes and high survivorship rates, measured as subsequent hip preservation procedures or conversion to THA.

The results demonstrated by the current study might indicate that residual or non-addressed impingement, either as cam, pincer or mixed morphology, was the indication for additional hip arthroscopy in PAO patients. However, treatment of these structural pathologies alone might be insufficient.

Further studies are needed to investigate treatment of the symptomatic dysplastic patient, specifically when to perform isolated PAO versus isolated hip arthroscopy or a combination of both procedures and whether these should be performed as one procedure. A multicenter randomized controlled trial investigating the topic of PAO with or without concomitant hip arthroscopy has been initiated [23]. The treatment algorithm described above by Edelstein *et al*. [25] is probably the most logical approach at the moment with promising results, but hopefully in the near future, studies will be able to demonstrate whether this strategy in the treatment of the dysplastic patient is resulting in better outcomes and patient satisfaction.

In summary, current knowledge demonstrates that the treatment of the symptomatic dysplastic patient is difficult and demanding. The treatment may consist of PAO as a minimum and maybe in combination with hip arthroscopy, if symptoms of IA pathology persist, either in combination or as an adjunct therapy in selected cases. In these cases, the patient might have the benefits of combined procedures where all IA pathologies are treated in combination with the reorientation of the acetabular coverage.

### Limitations

Several limitations exist in this study: first, a low completeness rate of PROM data. This is a common issue with data from registries, but completeness rate (68% at baseline and 52% at 2-year follow-up) in the present study is consistent with other registry studies [27]. In a validation study for the DHAR, Poulsen *et al*. demonstrated a high completeness rate in reported surgical data and lower completeness for PROM data, but with no significant difference between responders and non-responders at baseline PROM [27]. Secondly, it was not possible to report radiological data before the patients received their PAO, such as CE angles and AI angles. Therefore, the degree of dysplasia pre-operatively, whether it was severe or mild dysplasia, was not documented. Furthermore, preoperative radiological examinations might have illustrated possible cam impingement (average alpha angle was 69.3^o^) prior to the reorientation of the acetabulum, leading to impingement and the need for additional hip arthroscopy. Thirdly, it was not reported whether the dysplasia was categorized as retroversion or traditional dysplasia with lack of anterolateral acetabular coverage. Patients with severe dysplasia or retroverted acetabulum might have more severe intraarticular damage. Fourthly, this study did not investigate the socio-economic status of the patients and patient characteristics, such as body mass index, psychiatric or physical disorders other than hip disorders that might affect the PROMs.

## CONCLUSION

The current study demonstrates, in contrast to an earlier published study on Danish patients, significant improvements in subjective patient-reported outcomes 2 years after hip arthroscopy in hip dysplastic patients previously treated with PAO. There are still unsolved treatment outcome issues as only 50% of these patients reached clinically relevant outcome improvements.

## Data Availability

All data generated or analyzed during this study are included in this published article.
